# "Hugh Woods, M.D.," and the Newspapers

**Published:** 1890-02-15

**Authors:** 


### Passing Topics.

### "HUGH WOODS, M.D.," AND THE NEWSPAPERS.

Most of us, in the course of our lives, have met with certain wilful and mischievous children, who, mistaking longsufferance for encouragement, proceed from one enormity to another, until at last they reach the limit of their victim's endurance, and bring upon themselves a sharp and well-merited castigation. The youthful gentleman, who, during the past few months, has put forth so strenuous efforts to connect his name in the public mind with the subject of hospital administration, boasts a style redolent of the nursery, and is so manifestly incapable of achieving all the mischief he contemplates, that his philippics have been accorded little beyond that good-humoured indulgence which is an admirable substitute for a stronger feeling.

But Dr. Hugh Woods, failing, apparently, to understand that silence does not necessarily imply attention, still less acquiescence, has continued to devote a liberal share of the leisure he appears able to command, to the writing of letters to various public journals, wherein virulent attacks have been made upon institutions which perhaps more than any others deserve well of the public, and most certainly are entitled to the regard and consideration of every one who boasts a medical education. We do not propose to offer much comment upon either the style or the matter of these effusions. Whatever importance has attached to them has been derived from their publication in respectable journals, and the more serious question is whether a responsible newspaper is justified in giving a place in its columns to statements reflecting gravely not only upon indispensable organizations, but upon the probity of their managers, when not a tittle of evidence in support of the charges made is either given or promised.

Had Dr. Hugh Woods received his due at the hands of the editors, we venture to think his communications would never have seen the light, and he could not have boasted in a manner characteristic at once of his pretensions and his logic —"my statements are unchallenged because they cannot be denied."

Encouraged by impunity, experience shows that there are no limits to audacity, and accordingly we find that Dr. Hugh Woods in his last letters to an evening contemporary ceases from attacking systems and societies, and devotes himself to formulating hearsay indictments of grave import against their officers.

To this ardent reformer, whose profound knowledge of metropolitan hospitals must have been rapidly attained, since we find that his medical qualification was acquired in Dublin as recently as 1886, all appears base, and the enormities with which he lightly charges the whole body of hospital workers are recorded in the *Echo* of the 29th or 30th ult. They are set forth upon the information of one described somewhat vaguely as "a gentleman" and "my correspondent," whose testimony we are called upon to accept the more readily for the curious reason that he is "unconnected with hospitals or the medical profession," while, adds Dr. Hugh Woods loftily, "I must decline to give my authority for the above statements, but I believe them to be true."

No wonder that a fellow-practitioner exclaims, "very few men who have an M.D. degree would have written such a letter," or that the *Echo*, in an outburst of highly creditable, if somewhat tardy impatience, gives the *coup de grâce* to this precocious pretender, and "declines to publish any more unverified and probably unverifiable charges against metropolitan hospitals." It is not too much to ask a similar resolve of every responsible journal. Agricola.

